# Supplementary figures and images for: Assessing soil bacterial community and dynamics by integrated high-throughput absolute abundance quantification
Source: PeerJ. 2018 Mar 14;6:e4514. doi: 10.7717/peerj.4514 (PMC5857175; doi:10.7717/peerj.4514)

Total bacterial 16S rRNA gene

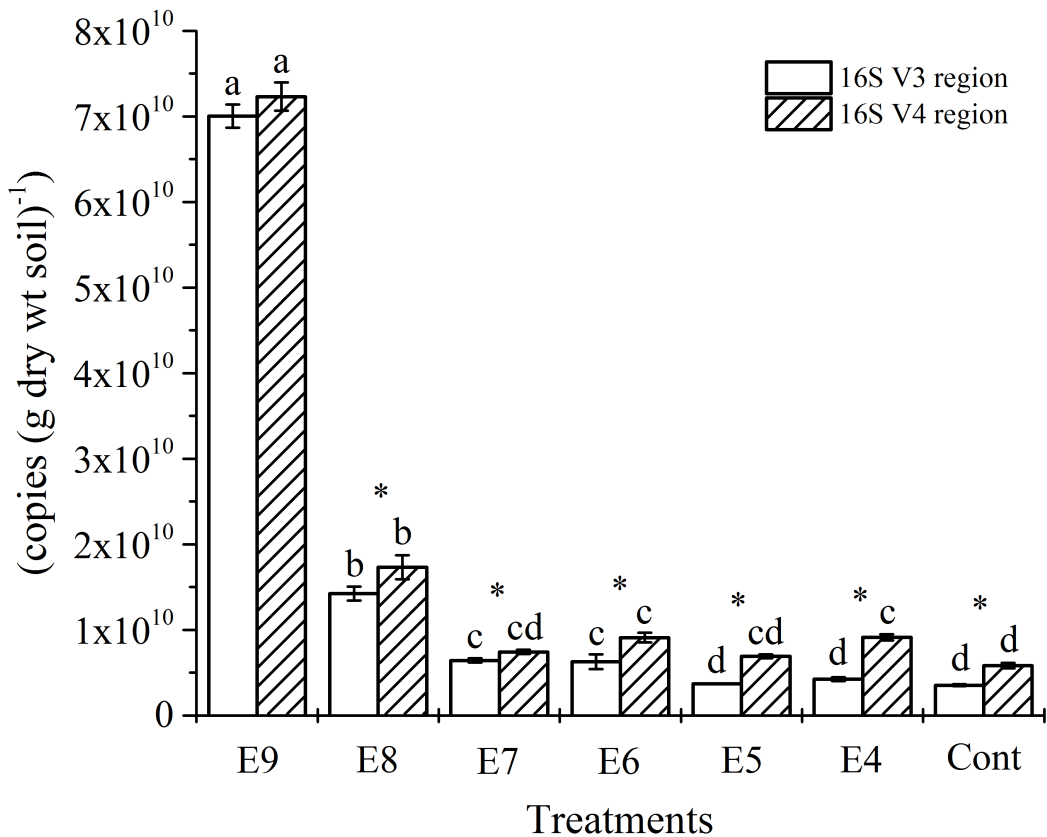

Supplement: Figure S1 — Treatments E9 to E4 are corresponding to the added internal reference strain EDL933 in soil with 6 different concentrations of 109 to 104 CFU (g dry wt soil)−1. Cont is the original control soil. Error bars represent the standard deviation of triplicate independent measurements. Hollow and shadow histograms, respectively, represent the total bacterial quantity quantified by V3 and V4 regions of 16S rRNA gene. Different letters indicate significant difference (p < 0.05) of V3 or V4 region of 16S rRNA gene between treatments detected by ANOVA test. * indicates significant difference between different regions of 16S rRNA gene. [file peerj-06-4514-s002.pdf]

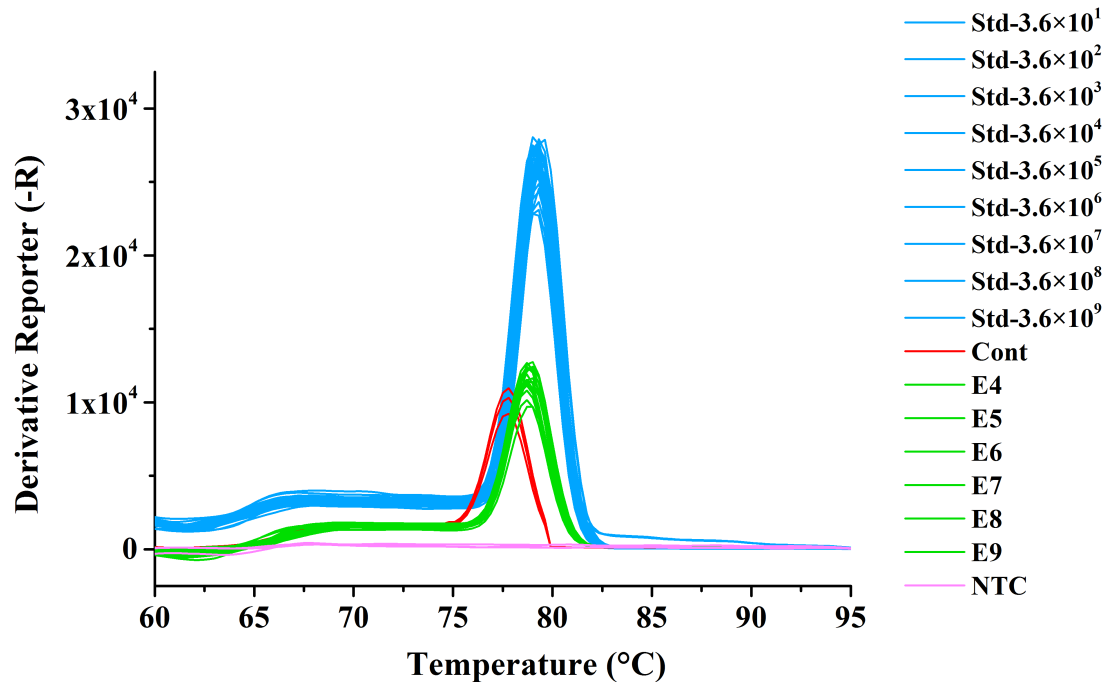

Supplement: Figure S2 — Std- 3.6 × 101 to Std-3.6 × 109 are the standard template DNA plasmid with 6 different concentrations of 3.6 × 101 to 3.6 × 10^9^ copies µL −1. E9 to E4 are the same as in Fig. S1, and the Cont is the original control soil. NTC is the no-temple control sample. [file peerj-06-4514-s003.pdf]

The detected *fliC* gene

(Log<sub>10</sub> copies (g dry wt soil)<sup>-1</sup>)

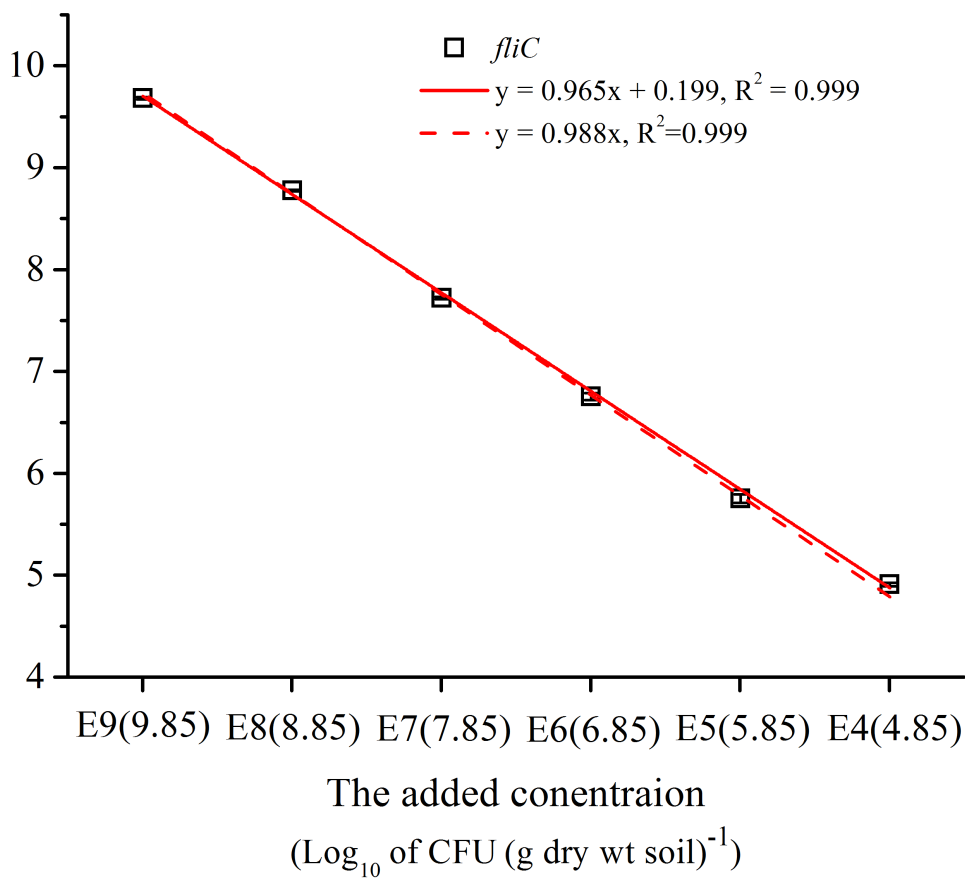

Supplement: Figure S3 [file peerj-06-4514-s004.pdf]

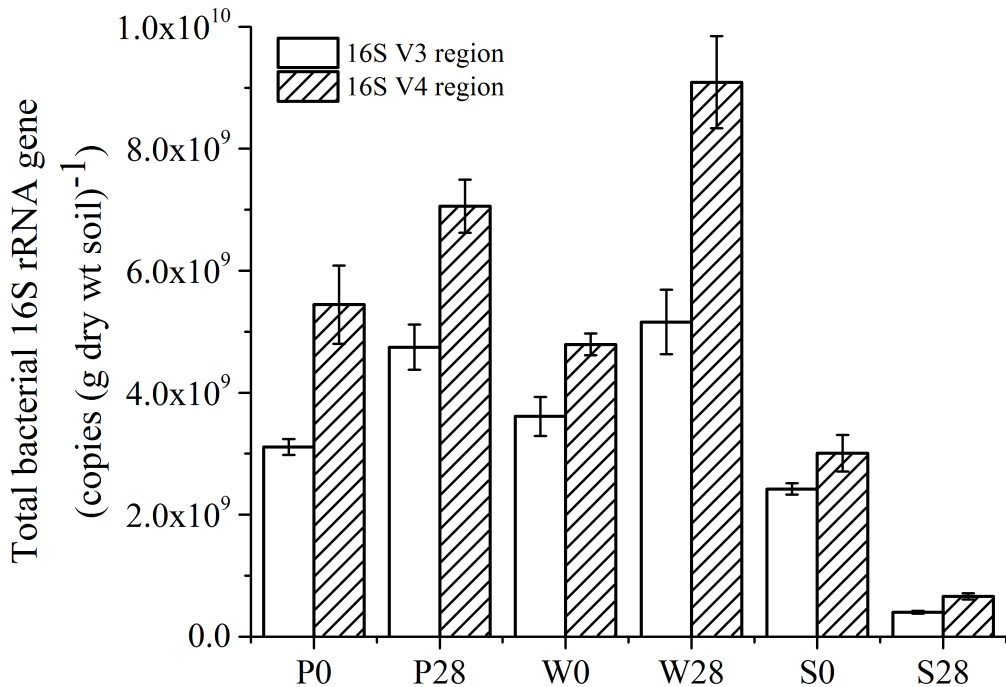

Supplement: Figure S4 — The P, S, and W treatments represent the soil added with PHE (100 µg (g dry wt soil)−1), NaN3 (0.1%, w/w), and PHE (100 µg (g dry wt soil)−1) and strain WG5 (approximately 1.00 × 10^7^ CFU (g dry wt soil)−1), respectively. 0 and 28 represent sampling at 0 and 28 days of incubation. Error bar represents the standard deviation of triplicate independent measurements. Hollow and shadow histograms represent the total bacterial quantity quantified by the V3 and V4 regions of 16S rRNA gene, respectively. [file peerj-06-4514-s005.pdf]

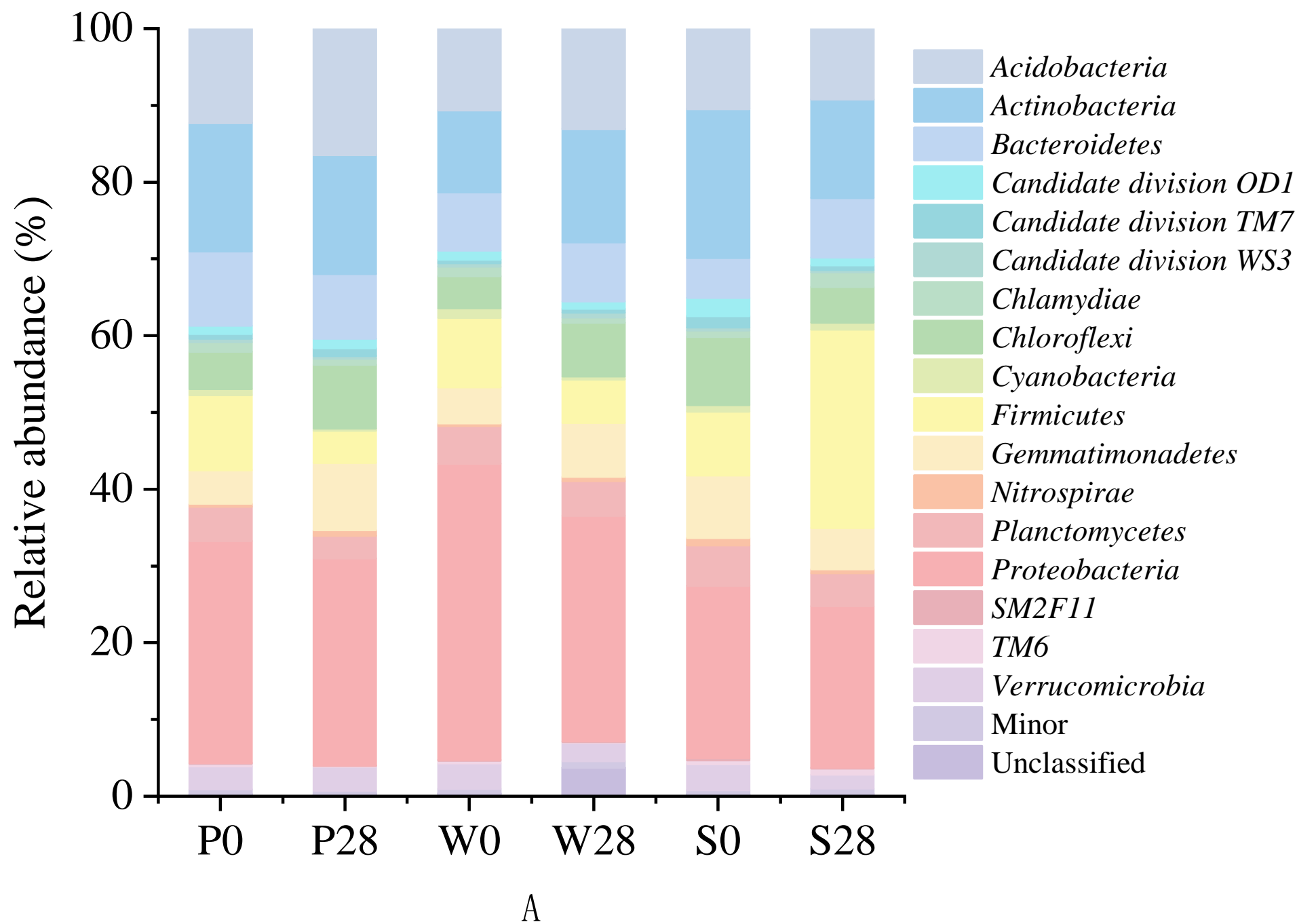

Supplement: Figure S5 — All classified phyla with a relative abundance of <0.1% in a sample were combined and reported as Minor. P0, P28, S0, S28, W0 and W28 are the same as in Fig. S4. [file peerj-06-4514-s006.pdf]

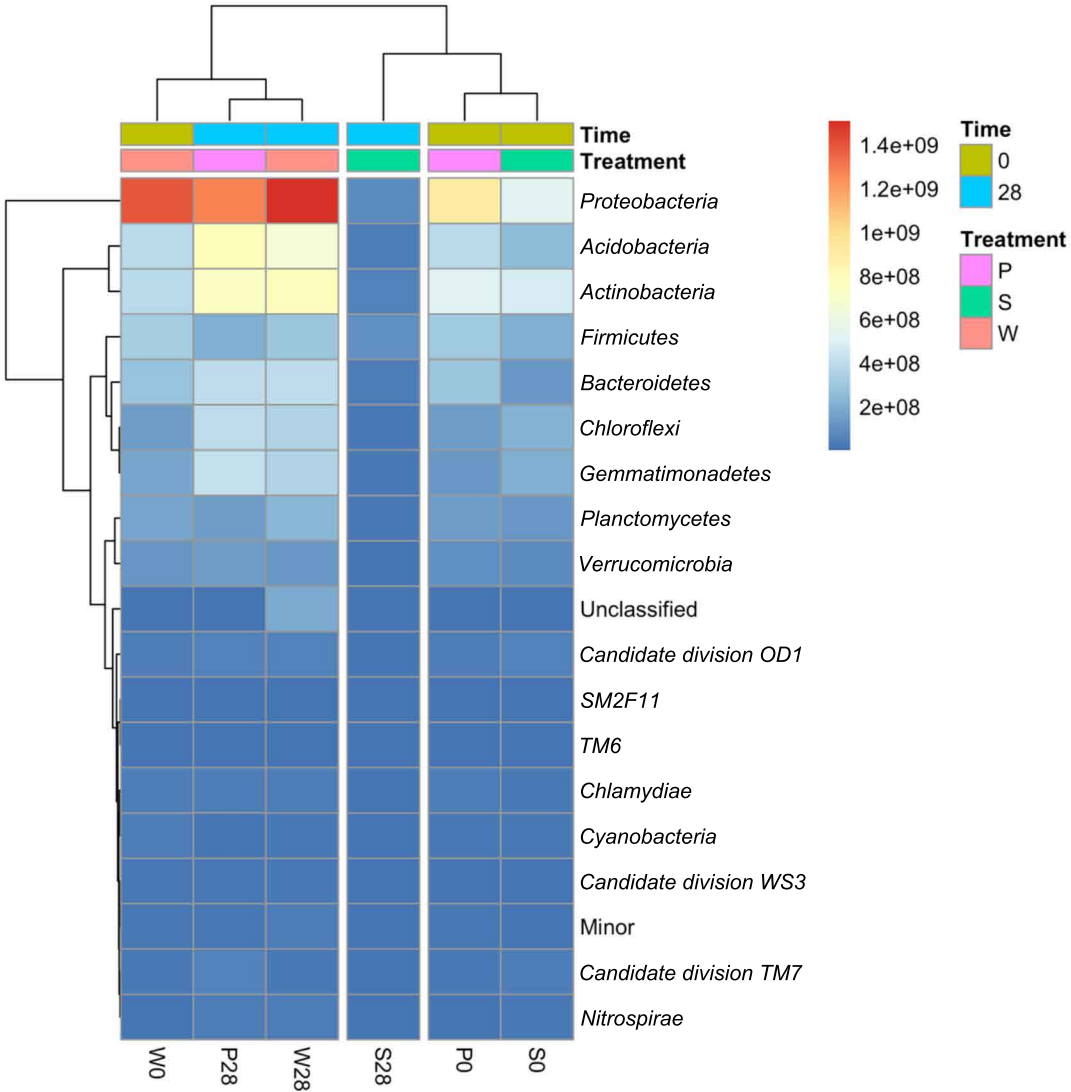

Supplement: Figure S6 — All classified phyla with a relative abundance of <0.1% in a sample were combined and reported as Minor. P0, P28, S0, S28, W0 and W28 are the same as in Fig. S4. The color code indicates absolute abundances, ranging from blue (low abundance) to red (high abundance). [file peerj-06-4514-s007.pdf]
